# Factors influencing the use of natural health products, in particular for concentration and cognition in Germany

**DOI:** 10.1186/s12906-024-04407-3

**Published:** 2024-02-27

**Authors:** Miriam Wolf, Agnes Emberger-Klein, Klaus Menrad

**Affiliations:** Department of Marketing and Management of Biogenic Resources, Hochschule Weihenstephan-Triesdorf (University of Applied Sciences), Technical University of Munich Campus Straubing for Biotechnology and Sustainability, Am Essigberg 3, D-94315 Straubing, Bavaria Germany

**Keywords:** Natural health product, Concentration and cognition, Decision making, CAM healthcare model, Value orientation

## Abstract

**Background:**

Natural health products (NHP) are an important part of the healthcare system. They are mainly non-prescription and sold over the counter, which requires active decision making by the consumer. Within the framework of the Complementary and Alternative Healthcare Model, this study aims to identify factors that influence NHP usage, in particular related to concentration and cognition (CC), a topic that concerns all ages and social classes within the population.

**Methods:**

Data were collected by means of a representative online survey (*n* = 1,707) in Germany in April 2022. Three user groups were defined: NHPCC users, who used NHP for CC (12 month prevalence); nCC-NHP users, who used NHP but not for CC indications (12 month prevalence); and past NHP users, who have used NHP but not within the previous 12 months. Independent influencing variables were categorized into predisposing, enabling, need, and health service use factors. Data were analyzed with descriptive statistics, inferential statistics, and binary logistic regression models to compare NHPCC users to nCC-NHP users (model 1) and to past NHP users (model 2).

**Results:**

A higher share of NHPCC and nCC-NHP users compared to past NHP users were women, self-medicated with NHP, and used information about NHP provided by health professionals or on product. Their openness-to-change value orientation was more pronounced than of past users. Compared to nCC-NHP and past NHP users, the probability of being an NHPCC user increased if an individual had more difficulties in daily attention and memory performance, made use of health professionals and literature to seek information about NHP, and used NHP for health support and illness prevention. Additionally, a female gender, NHP self-medication, and having higher values of self-transcendence were significant indicators for NHPCC usage compared to past NHP usage.

**Conclusion:**

NHP manufacturers, health professionals, and policymakers should be aware of the factors that lead to NHP consumption decisions and consider them in the development and optimization of healthcare strategies as well as in the marketing and communication strategies of companies producing NHP, in particular for CC. The current study can contribute to characterizing the target groups and to defining the aims and communication channels of such campaigns.

**Supplementary Information:**

The online version contains supplementary material available at 10.1186/s12906-024-04407-3.

## Background

The use of natural health products (NHP), including herbal medicine (HM) and natural nutrition supplements, is traditional in many cultures and is an important element in many healthcare systems [1–3]. NHP are part of complementary and alternative medicines (CAM). High prevalence rates demonstrate their widespread use, for example, a 12-month HM-usage prevalence rate of 75% among the German population in 2018 [4].

Often, sick individuals use NHP as a complement or alternative to conventional therapies for the treatment or management of symptoms [5–8]. This is especially the case when conventional pharmacological therapies cannot guarantee success. This applies, for example, to dementia, a major cognitive disorder. Dementia affects more than 55 million people worldwide, most of whom are more than 65 years old. Symptoms include cognitive decline and memory loss that impact a patient’s daily individual and social life [9]. Next to ill individuals, healthy individuals use NHP to support their health or to prevent illness [4, 5]. For example, increasing pressure, stress, and growing demands at work, university, and school encourage healthy adults and students to use products for cognition enhancement [10, 11]. Education and occupation, determinants of social class, were found to be indicators for Alzheimer diseases [12]. They are part of public health interventions targeting dementia [13, 14], as lower education and its socioeconomic consequences were found to be a significant risk factor for dementia [12, 15]. On the other hand, subjective memory complaints were found to be associated with higher education, which could be due to more noticeable changes in memory performance [16, 17]. However, associations between subjective memory declines and education are weak [18]. Difficulties in daily attention and memory performance were found weaker for individuals with less than 10 years of school education as well as for those with a university degree compared to people with more than 10 years of school education but without a university degree [19]. Thus, concentration and cognition issues affect people of all ages and social classes.

The market offers a variety of NHP for concentration and cognition (NHPCC) enhancement [20]. The mechanisms of NHPCC reach from preventing brain cells from free radicals and oxidative stress to improving neuron functions, increasing blood circulation, and providing energy or precursors to neurotransmitters [21]. Gingko biloba, for example, is a plant traditionally known for the treatment of cognition issues, including problems with memory and concentration [22].

Many consumers perceive NHP as effective and safe, have little to no concern about potential risks like side effects, and prefer them to conventional medicine [23, 24]. A preference for NHP can, for example, source from positive experiences with NHP, trust due to family traditions [4], and the feeling of treatment control [25]. Most NHP are non-prescription and sold over the counter (OTC) [26]. Self-medication with NHP is a common practice. Few users inform their practitioners about self-medicated NHP applications [4, 23, 27]. For example, in 2018, 92% of German users consumed HM via self-medication, and only 38% consulted their practitioners about this practice [4]. Not visiting a practitioner for non-fatal issues like the desire to enhance concentration and cognition and to instead practicing self-medication can reduce the burden on the healthcare system in terms of capacity and financial aspects [28, 29]. Pharmacists are a popular source for information about OTC drugs, including NHP [30, 31]. Further, the healthcare system in the twenty-first century has shifted from traditional disease-focused treatment approaches to a patient-centered model that focuses on support and care [32, 33]. Shared decision making involves the consumer of medical products in health care decisions and gives more responsibility for active self-informing [34].

The better a health service or product is in considering and meeting important values of the individual, the more likely the individuals will make use of it [35, 36]. Values were found to substantially impact consumer behavior in various situations [37, 38], including health and medical decision making. Schwartz (2012) [39] defined values as specific situation-exceeding beliefs directed toward desirable goals. They strongly link to affect and motivate action by serving as criteria or standards in evaluation and selection processes. Schwartz’s Theory of Basic Values includes ten core values that individuals order and prioritize according to their relative importance, and that can be summarized in four main value orientations: Self-enhancement, self-transcendence, conservation, and openness-to-change [39, 40]. Understanding value orientations in health-related decision making and taking them into account can contribute to better communication between health professionals and patients [35, 41, 42].

**Fig. 1:**
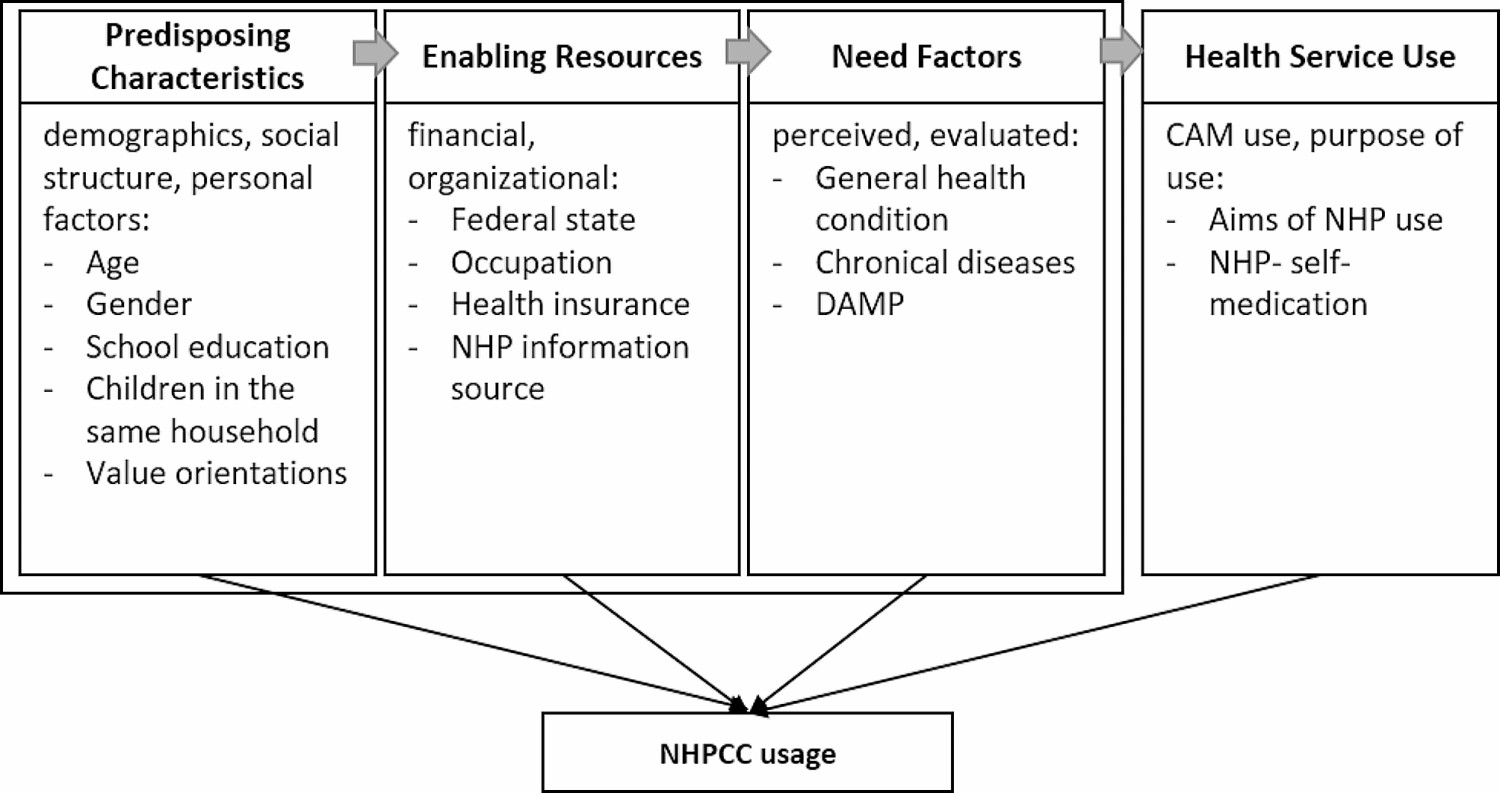
Conceptual framework of the study: Andersen’s behavioral model of health services use, modified on the basis of the CAM healthcare model by Fouladbakhsh and Stommel (2007). DAMP = Difficulties in daily attention and memory performance

Decision making in health services underlies a complex process that involves multiple factors. Important models in this field include at least two kinds of variables for predicting behavior: The value an individual places on a specific goal (e.g., push or pull factors) and the association/expectation that a particular action will lead to reaching this goal [43]. Following this principle, Andersen’s healthcare utilization model (AHUM) [44, 45] distinguishes between three major factor categories that predispose, enable, or suggest a need to use health services. *Predisposing factors* include sociodemographics such as gender or age as well as health beliefs and mental factors like values and attitudes toward health services. *Enabling factors* are resources and conditions that serve to enable health service utilization. They include financial factors (e.g. health insurance), and organizational factors, for example, the type of used healthcare sources, such as consulting practitioners. *Need factors* can be self-perceived (e.g., an individual’s own experienced health status) or evaluated (e.g., objective measurements, professional assessments) [46]. The CAM Healthcare Model (CAMHM) by Fouladbakhsh and Stommel (2007) expanded the AHUM by including health service use variables specifically regarding CAM usage, such as self-directed CAM practice and CAM products. The CAMHM aims to identify key factors that are especially related to CAM. It was used as a conceptual framework for the present study (see Fig. 1).

To provide better insights into healthcare decision making related to NHP usage, several studies have called for empirical testing of CAM healthcare models [47, 48] based on survey data and focusing on an individual’s behavior related to health along with factors influencing CAM usage [33, 49]. In this sense, research is needed to identify key psychological, sociocultural, and economic factors that affect consumer decisions regarding health activities and treatment choices [33]. Thus, one aim of this study is to analyze prevalence rates for NHP usage in Germany and relevant predisposing, enabling, need, and health service use (HSU) factors that explain NHP usage in the German population. In addition, the study examined those factors that differentiate between different groups of NHP users in Germany, taking into account the CAMHM as a research framework.

Previous studies have shown that healthcare decision making depends on the indication or targeted disease. Accordingly, the likelihood of CAM usage decisions varies depending on specific indications and diseases [4, 50, 51]. Many previous studies have focused on either one specific disease, such as cancer [52], on one specific consumer group, such as type 2 diabetes patients [53], or on a specific subpopulation, like CAM use in African Americans [54]. However, the use of NHPCC has not been intensively analyzed, even though dementia and concentration problems are increasing in many populations worldwide [9, 55–57]. Therefore, another target of this study is to identify factors that are characteristic for different groups of NHP users with a special focus on NHP for concentration and cognition (NHPCC).

The data for our study were collected using a representative sample of the German population. The data allow further insights into NHP use and its influencing factors in general and in the under-researched field of NHPCC. Insights provided by this study are valuable for health policymakers and administrators, who can use them to develop new or optimize existing public health strategies, including NHPCC. Further, a deeper knowledge of NHP and NHPCC user characteristics and behaviors can support companies in this field in developing targeted business strategies. It can also support health professionals to better understand the behavior of NHP or NPHCC users in Germany and to adapt consultations on NHP and treatment recommendations in shared decision making with their patients. The current study can also contribute to characterizing the groups to target with health campaigns in the private and public sector and to define the information sources and aims of such campaigns.

## Methods

A cross-sectional online survey using a standardized questionnaire was conducted in April 2022 in Germany. Participation criteria were a minimum age of 18 years, a residence in Germany, and sufficient German language skills. A market research institute recruited the participants via an online panel. Quota according to sociodemographic characteristics (age, gender, federal state, and residence size) ensured the survey was representative of these variables for the general German population (age 18 years +). Immediately before starting the questionnaire, participants received information about the purpose of the study, including the theme of NHP, and agreed to the privacy policy. After finishing, they received a monetary incentive of one Euro from the market research institute. Participation was voluntary. The questionnaire was completed by 2,207 individuals. We carefully screened the data according to Schendera 2011 [58], excluding straight-liners, who had mostly or always chosen the same response option, and speeders, who finished the questionnaire in less than half of the median time of the whole sample. The remaining reliable and valid dataset included 1,707 participants. Additionally, participants who had never used NHP were excluded from this study, because the complete first part of the questionnaire could not be answered by these people. Therefore, a total of 1,464 individuals, those who reported general NHP usage experience, remained for further data analyses. An allowable margin error of < 5% permits this sample size to be sufficient to obtain adequate results for the objective of this research [59]. This study received approval from The Ethic Commission of the Faculty of Medicine, Technical University of Munich, on April 2, 2022.

### Questionnaire design

The questionnaire had two main parts. Items and scales were based on established survey instruments like the Short Schwartz’s Value Survey (SSVS) [60] or were derived from literature.

After quota-sampling questions at the beginning, the first section identified NHP users. They all answered the first part of the questionnaire, which was focused on NHP usage and consumption behavior. It contained, for example, questions about the aims of NHP usage (support/maintain health; illness prevention; treatment of disease/symptoms) and about NHP self-medication (yes; no). The question about the application fields of NHP was later used to identify NHPCC users.

The second part of the questionnaire contained questions about general health status and health behavior. An Attention and Performance Self-Assessment (APSA) was then applied to assess everyday concentration and cognition. Participants evaluated how often 20 described situations happened to them within the past four weeks based on a five-point Likert scale from “never” [1] to “always” [5, 19]. Continuing with the SSVS [61], participants evaluated its 10 core values on a seven-point Likert scale from “against my principles” (-1), “not important to me at all” (0) to “very important to me” [5]. At the end of the questionnaire, we asked for general sociodemographic data. An excerpt of the questionnaire that includes all parts relevant to this study is provided in supplementary file 1.

### Data analyses

For this study, participants were categorized into three main groups according to their experience with NHP. Past NHP users had not taken NHP in the previous 12 months but had taken it previously. NHPCC users reported NHP usage in the previous 12 months explicitly for at least one indication out of the following: concentration and cognition, dementia, headache and migraine [62], and tinnitus [19, 63–65]. NHPCC users could additionally take NHP in other application fields. nCC-NHP users reported NHP usage within the previous 12 months but not for indications that directly affected concentration and cognition as listed for NHPCC users. Due to the sample size, a normal distribution can be assumed. Descriptive statistics (frequencies, mean and SD values, percentages) were used to describe the predisposing, enabling, need, and health service use factor characteristics for the whole NHP-user sample and each group. We performed Chi-square and z-tests with *p*-values adjusted with the Bonferroni method for comparing column proportions implemented in SPSS to determine significant relationships between the variables and the three user groups (NHPCC users, nCC-NHP users, past NHP users). Significance tests were performed on a *p* < 0.05 level. To determine effect sizes, we calculated Cramer’s V.

Variables were categorized into predisposing, enabling, and need factors according to the definitions and suggestions by Andersen and Davidson (2001) [66] and Babitsch et al. (2012) [46]. As recommended by Fouladbakhsh and Stommel (2007), we extended the initial behavioral model with health service use factors [47]. Figure 1 shows the conceptual framework of this study based on the AHUM and modified on the basis of the CAMHM. The figure presents all elements of each factor category applying to this study.

Predisposing factors were age, gender, school education, children living in the same household, and value orientations of the respondents. To investigate value orientations, we analyzed the SSVS according to the suggestions by Boer (2013), who defined the value orientation as the mean of the ratings of the associated values: Self-enhancement represents the mean of the ratings of the single values of achievement and power; the mean ratings of universalism and benevolence represent self-transcendence; conservation is the mean of the ratings of the values of security, conformity, and tradition; and openness-to-change is the mean of the ratings of stimulation, self-direction, and hedonism [60]. Levene statistics validated the assumption of homogeneity of variances (*p* > 0.05) for value orientations and the general health condition. Thus, as suggested by Field [67], we performed an ANOVA for group comparisons to test for significant differences between the user groups, the Hochberg GT2 test for post-hoc comparisons, and calculated ω² for effect sizes.

Federal state (east/west), health insurance (public/private), occupational status (employed/not employed), and information sources (health professionals, e.g., practitioners and pharmacists; online and social media; literature and journals; family and friends; contained on product (e.g., declaration and package insert), were included in this survey as enabling factors. The occupation status “not employed” included pensioners, students, unemployed jobseekers, and non-workers. The need factors that were analyzed were self-reported general health condition, whether one suffers from chronic diseases, and the results of the APSA. The APSA was analyzed following Bankstahl and Görtelmeyer (2013). Mean and standard deviation were calculated for all 20 situation statements and were also scored overall for each user group to determine difficulties in daily attention and memory (DAMP) [19]. For group comparisons of the APSA regarding DAMP, we performed Welch`s ANOVA and Games-Howell tests for post-hoc comparisons because the assumption of homogeneity of variances was violated. As recommended by Field [67], in such cases, these statistical methods are more robust than traditional ones. We used ω² for the effect size calculation.

Health service use factors included in this study were the aims of NHP use (support and maintenance of health/prevention of illness/treatment of diseases or symptoms) and self-medication with NHP (yes/no).

Multicollinearity and outliers checks for all independent variables showed no problem in the data [68, 69]. Two binary logistic regressions were performed to determine whether and, if so, which predisposing, enabling, need, and health service use factors were indicators for NHPCC usage as distinguished from nCC-NHP usage (model 1) and from past NHP usage (model 2). Due to the high difference in group sizes (NHPCC user *n* = 236; nCC-NHP user *n* = 993), we used a random sample of about half of the nCC-NHP user group to estimate regression model 1.

All data were analyzed using IBM SSPS Statistics 29.0.0 for Windows.

## Results

The sample included 1,707 participants, of which 1464 (85.8%) have ever used NHP and thus were further analyzed for this study. Of these 1464 participants, 16.1% did not use NHP within the previous 12 months (*n* = 235; past NHP user); another 16.1% consumed NHP for CC indications (*n* = 236; NHPCC user); and 67.8% (*n* = 993; nCC-NHP user) used NHP but not for CC indications. In sum, the 12-month prevalence of NHP usage was 71.9% (*n* = 1,229; NHPCC user + nCC-NHP user).

### Predisposing factors

Table 1 shows frequencies and results of Chi-square tests for the categorical predisposing factors.

**Table 1:** Predisposing factors (categorical variables), *n* = 1,464

Variables	Total sample *n* = 1,464	NHPCC user, *n* = 236 (1)	nCC-NHP user, *n* = 993 (2)	Past NHP-user, *n* = 235 (3)	X²(df); *p*-value	z-test (Bonferroni adjusted)	Cramer’s V
Total (%)	100	16.1	67.8	16.1			
**Gender**							
Male	43.2	38.6	39.8	62.1	X² (2) = 41.13, *p* = < 0.001	1|3; 2|3	0.168
Female	56.8	61.4	60.2	37.9			
**Age**							
18‒29 (young)	15.6	19.9	15.8	10.6	X² (4) = 18.23, *p* = 0.001	1|3	0.079
30‒59 (middle-aged)	50.6	55.9	49.8	48.5			
60+ (senior)	33.7	24.2	34.3	40.9		1|2; 1|3	
**Education (years)**							
< 12 years	47.8	47.0	46.1	55.7	X² (2) = 7.12, *p* = 0.028	2|3	0.070
>= 12 years	52.2	53.0	53.9	44.3			
**Children in household**							
No	79.2	73.3	79.5	84.3	X² (2) = 8.67, *p* = 0.013	1|3	0.077
Yes	20.8	26.7	20.5	15.7			

Chi-square tests proved significant associations for all categorical predisposing variables and the user groups (*p* < 0.05), and Cramer’s V indicated small effect sizes (< 0.3) [67]. The proportion of female NHPCC users (61.4%) and nCC-NHP users (60.2%) was significantly higher than that of female past NHP users (37.9%). NHPCC users were more often young (19.9%) and less often seniors (24.2%) than past NHP users (young 10.6%; senior 40.9%). More than half of all NHPCC and nCC-NHP users had at least 12 years of school education ( > = 53.0%), and 26.7% of NHPCC users had children living in the same household, which is higher than for nCC-NHP users (20.5%) or past NHP users (15.7%).

Table 2 shows the mean values of value orientations for the three groups and the results of group comparison tests.

**Table 2:** Predisposing factor of value-orientations (metric variables); means and group comparison, *n* = 1,464

Variables	Total sample	User Group Means (SD)			ANOVA (F-Ratio)	Hochberg GT2 post-hoc	ω²
		NHPCC user (1)	nCC-NHP user (2)	Past NHP user (3)			
Total (n)	1,464	236	993	235			
Openness-to-change	2.92 (0.99)	3.02 (1.05)	2.95 (0.96)	2.70 (0.98)	15.16***	1|3; 2|3	0.01
Conservation	3.17 (1.00)	3.15 (1.03)	3.17 (0.99)	3.18 (1.03)	0.09		
Self-Transcendence	3.93 (0.97)	4.01 (0.99)	3.95 (0.98)	3.78 (0.90)	7.21*	1|3; 2|3	> 0.01
Self-Enhancement	1.50 (1.26)	1.59 (1.32)	1.49 (1.27)	1.43 (1.15)	3.08		

Scale: -1 against my principles, 0 not important at all, 1 not important, 2 rather not important, 3 rather important, 4 important, 5 of supreme importance; ***p ≤ 0.001; *p ≤ 0.05; post-hoc significant differences between: 1 = NHPCC user group, 2 = nCC-NHP user group; 3 = Past NHP user group; SD = standard deviation

Overall, self-transcendence was rated as the most important, followed by conservation and openness-to-change. Self-enhancement was least important for all user groups. Except conservation, all value orientations were the most important for NHPCC users, followed by nCC-NHP users, and the least important for past NHP users. The Hochberg GT2 post hoc test showed significant differences in the value orientations between past NHP users and both NHP-using groups (NHPCC users, nCC-NHP users) for openness-to-change and self-transcendence. Omega square values were close to 0, thus indicating negligible effect sizes.

### Enabling and need factors

The frequencies and results of Chi-square tests for binary-coded enabling and need factors are shown in Table 3.

**Table 3:** Enabling and need factors, *n* = 1,464

	Variables	Total sample *n* = 1,464	NHPCC user, *n* = 236 (1)	nCC-NHP user, *n* = 993 (2)	Past NHP-user, *n* = 235 (3)	X²(df); *p*-value	z-test (Bonferroni adjusted)	Cramer’s V
	Total (%)	100	16.1	67.8	16.1			
Enabling factors	**Federal State**							
	East	14.9	15.7	15.5	11.5	X² (2) = 2.56, *p* = 0.278		0.042
	West	85.1	84.3	84.5	88.5			
	**Occupation**							
	Employed	65.6	72.5	64.9	61.7	X² (2) = 6.74, *p* = 0.034	1|3	0.068
	Not employed	34.4	27.5	35.1	38.3			
	**Health insurance**							
	Public	75.9	76.3	76.7	71.9	X² (2) = 2.44, *p* = 0.296		0.041
	Private	24.1	23.7	23.7	28.1			
	**NHP Information source**							
	Health professional	55.5	63.6	55.3	48.1	X² (2) = 11.45, *p* = 0.003	1|3; 2|3	0.088
	Online/social media	29.2	40.7	28.2	21.7	X² (2) = 21.93, *p* = < 0.001	1|2; 2|3	0.122
	Literature/journals	25.0	39.8	24.1	14.0	X² (2) = 43.79, *p* = < 0.001	1|2; 1|3; 2|3	0.172
	Family/friends	36.2	41.5	35.8	32.8	X² (2) = 4.19, *p* = 0.123		0.053
	On product	48.4	57.6	50.1	31.9	X² (2) = 34.70, *p* = < 0.001	1|3; 2|3	0.154
Need factor	**Chronic diseases**							
	yes	54.4	56.4	56.2	44.7	X² (2) = 10.60, *p* = 0.005	1|3; 2|3	0.085

No significant differences between the user groups were found for the federal state and health insurance. Of the participants, 75.9% had public health insurance. The share of employed individuals was significantly higher in the NHPCC user group (72.5%) than in the past NHP user group (61.7%). Chi-square tests proved significant associations for all NHP information sources despite family/friends and the user groups (*p* < 0.05). Effect sizes were small (Cramer’s V < 0.3) [67]. Health professionals were the information source that was most mentioned by all groups (average 55.5%), but it was significantly less mentioned by past users compared to NHPCC and nCC-NHP users. Second most mentioned was information on the product itself (declaration, package insert). The share of people in each category for all information sources was highest among NHPCC users, followed by nCC-NHP users, with the least past NHP users.

The share of individuals who suffered from at least one chronic disease was around 56% and significantly higher within the NHP user groups (NHPCC and nCC-NHP users) than within the past NHP user group (44.7%).

Table 4 presents the results of the metric need factors, including the general health condition and the results of the APSA, as well as the results for group and post-hoc group comparisons.

**Table 4:** Need factors (metric variables), *n* = 1,464

Variables	Total sample	User Group Means (SD)			ANOVA (F-Ratio)	Games-Howell post-hoc	ω²
		NHPCC user (1)	nCC-NHP user (2)	Past NHP user (3)			
Total (n)	1,464	236	993	235			
General health condition	2.40 (0.79)	2.37 (0.79)	2.43 (0.80)	2.34 (0.77)	1.77		
APSA	1.17 (0.58)	1.34 (0.62)	1.18 (0.58)	0.98 (0.51)	15.57***	1|3; 1|2; 2|3	0.03

Scale: general health condition 1 = very good, 2 = good, 3 = fair, 4 = bad, 5 = very bad; APSA 0 = never, 1 = seldom, 2 = sometimes, 3 = often, 4 = always; ***p < 0.001; post-hoc significant differences between: 1 = NHPCC user group, 2 = nCC-NHP user group; 3 = past NHP user group; SD = standard deviation

All user groups evaluated their general health condition as rather good. There are no significant differences between the groups concerning general health condition (*p* > 0.05).

Past NHP users indicated the least difficulties in daily attention and memory performance (DAMP) (APSA = 0.98). NHPCC users indicated the highest difficulties in daily attention and memory performance with an APSA value of 1.34, a value indicating DAMP between seldom and sometimes. The Games-Howell post-hoc test showed significant differences between all user groups for the APSA results (*p* < 0.05). The effect size with an omega square value of 0.03 is marginal.

### Health service use

Descriptive data and the results of Chi-square tests for the health service use variables for the different NHP user groups are presented in Table 5.

**Table 5:** Health service use variables regarding NHP utilization, *n* = 1,464

Variables	Total sample *n* = 1,464	NHPCC user, *n* = 236 (1)	nCC-NHP user, *n* = 993 (2)	Past NHP-user, *n* = 235 (3)	X²(df); *p*-value	z-test (Bonferroni adjusted)	Cramer’s V
Total (%)	100	16.1	67.8	16.1			
**Aims**							
Support/maintain health	76.6	90.7	77.4	59.1	X² (2) = 66.49, *p* = < 0.001	1|3; 1|2; 2|3	0.213
Prevention of illness	50.1	69.1	51.1	26.8	X² (2) = 85.32, *p* = < 0.001	1|3; 1|2; 2|3	0.241
Treatment of symptoms/diseases	71.4	70.8	73.2	64.3	X² (2) = 7.52, *p* = 0.023	2|3	0.072
**NHP self-medication**							
Yes	83.6	89.0	87.0	63.8	X² (2) = 80.43, *p* = < 0.001	1|3; 2|3	0.234

Subscript letters denote a subset of user groups whose proportions do not differ significantly from each other at a *p* ≤ 0.05 level

For the NHPCC and nCC-NHP user group, supporting or maintaining health was the most often mentioned aim of NHP consumption (NHPCC 90.7%; nCC-NHP 77.4%). The proportions differed significantly between all user groups for the aim of health support and illness prevention. The share of individuals whose aim for NHP consumption was the treatment of diseases/symptoms was highest in the nCC-NHP user group (73.2%). More current NHP users (NHPCC 89.0%; nCC-NHP 87.0%) than past NHP users (63.8%) took NHP self-medicated. Effect sizes were small (Cramer’s V < 0.03) [67].

### Results of the binary logistic regression models

To examine the influence of predisposing, enabling, need, and health service use factors on NHPCC as distinguished from nCC-NHP usage (model 1) and past NHP usage (model 2), we conducted two binary logistic regression models. The results are shown in Table 6.

**Table 6:** Results of the binary logistic regression models to identify factors for NHPCC usage as distinguished from nCC-NHP usage (model 1) and past NHP usage (model 2)

Variables			Model 1: nCC-NHP user (*n* = 492) vs. NHPCC user (*n* = 236)					Model 2: Past NHP user (*n* = 235) vs. NHPCC user (*n* = 236)				
					95% CI					95% CI		
			B	OR	Lower	Upper	R²	B	OR	Lower	Upper	R²
Predisposing factors		Age: middle-aged (young = 0; middle-aged = 1)	-0.28	0.75	0.46	1.24		0.11	1.12	0.51	2.45	
		Age: age old (young = 0; old = 1)	-0.61*	0.54	0.30	0.98		-0.01	0.99	0.41	2.40	
		Gender (male = 0; female = 1)	-0.12	0.89	0.61	1.29		**0.84** **	2.31	1.35	3.95	
		School education (< 12years = 0; > 12 years = 1)	-0.19	0.82	0.58	1.18		0.06	1.06	0.62	1.83	
		Children in the same household (no = 0; yes = 1)	0.26	1.30	0.85	2.00		0.45	1.57	0.80	3.07	
	Value orientation	Openness-to-change	-0.04	0.96	0.78	1.17		0.15	1.16	0.87	1.55	
		Conservation	-0.06	0.94	0.78	1.14		-0.43**	0.65	0.50	0.85	
		Self-enhancement	0.03	1.03	0.89	1.19		0.06	1.07	0.84	1.35	
		Self-transcendence	0.13	1.14	0.92	1.41	0.030	0.35**	1.42	1.05	1.91	0.132
Enabling factors		Federal state (west = 0; east = 1)	-0.06	0.94	0.59	1.50		0.45	1.57	0.73	3.39	
		Occupation (not employed = 0; employed = 1)	0.19	1.20	0.79	1.84		0.46	1.58	0.84	2.97	
		Health insurance (public = 0; private = 1)	0.04	1.04	0.70	1.54		-0.16	0.85	0.48	1.51	
	Information source	Health professional (no = 0; yes = 1)	0.40*	1.49	1.04	2.14		1.07***	2.92	1.69	5.04	
		Online/Social media (no = 0; yes = 1)	0.35	1.41	0.98	2.03		0.42	1.52	0.86	2.67	
		Literature/Journals (no = 0; yes = 1)	0.86***	2.36	1.63	3.41		1.15***	3.17	1.74	5.77	
		Family/friends (no = 0; yes = 1)	0.08	1.08	0.76	1.54		0.02	1.02	0.60	1.75	
		On product (no = 0; yes = 1)	0.12	1.13	0.79	1.61	0.110	0.40	1.49	0.87	2.52	0.347
Need factors		General health condition	-0.04	0.96	0.74	1.23		0.01	1.01	0.68	1.49	
		Chronic diseases (no = 0; yes = 1)	0.09	1.09	0.75	1.59		0.24	1.27	0.72	2.25	
		APSA: DAMP	0.45**	1.57	1.15	2.15	0.127	0.97***	2.63	1.58	4.37	0.403
Health service use	Aims	Support/maintain health (no = 0; yes = 1)	0.64**	1.91	1.12	3.24		1.40***	4.04	2.05	7.94	
		Prevention of illness (no = 0; yes = 1)	0.58**	1.78	1.24	2.57		1.60***	4.97	2.90	8.52	
		Treatment of symptoms/diseases (no = 0; yes = 1)	-0.26	0.77	0.52	1.14		-0.01	0.99	0.57	1.73	
		NHP self-medication (no = 0; yes = 1)	-0.09	0.91	0.53	1.58	0.165	1.38***	3.96	2.07	7.59	0.563

B = regression coefficient b; OR = odds ratio. CI = confidence intervals for odds ratio; R²= Nagelkerke Pseudo R². *p ≤ 0.05; **p ≤ 0.01; ***p ≤ 0.001. Bold data are significant on a 0.05 level

Likelihood ratio tests showed a statistical significance of *p* < 0.001, indicating that both regression models predict the dependent variable better than the respective null model. Non-significant results of the Hosmer-Lemeshow test across all factor blocks assessed reasonable fits of goodness for both models. Pseudo-R² statistics showed a reasonable fit and represented decent-sized effects for distinguishing between the groups in the two models (Nagelkerke model 1: R² = 0.165; model 2: R² = 0.563) [67]. In both models, the enabling block improved the Nagelkerke R² the most (model 1: 8%; model 2: 21%). In model 1, the overall correct classification rate was 71%. The correct classification rate in model 2 was 81.7%.

In model 1, predisposing factors explained 3% of variance. The only predisposing factor that significantly decreased the odds of being an NHPCC user compared to an nCC-NHP user was old age. After entering enabling factors, the explained variance rose to 11%. The odds of being an NHPCC user increased when health professionals (factor 1.49) or literature/journals (factor 2.36) were used for NHP information seeking. For every one-unit increase in DAMP, which represents a need factor, the odds of being an NHPCC user increased about 1.57 times. In addition, the odds of being an NHPCC user increased when NHP were taken to support health (factor 1.91) and for illness prevention (factor 1.78). The HSU block improved the Nagelkerke R² from 0.127 to 0.165.

In model 2, being female increased the odds of being an NHPCC user by 2.31 times compared to being a past NHP user. For a one-unit decrease in the importance of conservation values, the odds of being an NHPCC user increased by 53.9%. In contrast, for a one-unit increase in the importance of self-transcendence values, the odds of being an NHPCC user increased by about 42%. Predisposing factors explained 13.2% of the variance in the dependent variable, while the addition of enabling factors raised the explained variance to 34.7%. The odds of being an NHPCC user significantly increased when health professionals (factor 2.92) and literature/journals (factor 3.17) were used as NHP information sources. An indication of more difficulties in daily attention and memory performance increased the odds of being an NHPCC user by about a factor of 2.63. After adding need factors and entering HSU factors to model 2, the explained variance rose from 40.3% up to 56.3%. Concerning HSU factors, the odds of being an NHPCC user increased when NHP were used for health support (factor 4.04) and illness prevention (factor 4.97) as well as when NHP were taken for self-medication (factor 3.96).

## Discussion

The 12-month prevalence rate in Germany for NHP in general was found to be high at 71.9%, which is only a little lower than the 12-month HM-prevalence rate of 75.4% found in 2018 [4]. In the group of NHP users, 16.1% took NHP for indications that are linked to concentration and cognition. These numbers show the relevance of NHP and NHPCC use in the German healthcare system.

In this study, we analyzed three different NHP user groups, namely past NHP users, nCC-NHP users, and with a special focus NHPCC users. Descriptive statistics determined predisposing, enabling, need, and health service use factors that significantly differed between single groups or between all three user groups.

Focusing on influencing factors for NHPCC usage as distinguished from the other groups, two logistic regression models found that enabling factors and health service use variables contributed the most to the explained variance. While predisposing and need factors contributed little (< 5%) to differentiate NHPCC usage from nCC-NHP usage (model 1), their contribution to explained variance between NHPCC and past NHP usage was more than 13% (model 2). However, in both models, correct classification rates were high (71% in model 1; 81.7% in model 2). Thus, the CAMHM modification of the AHUM is a useful theoretical framework to explain the use of CAM products, such as NHP. Taking into account the R^2^-values and the classification rates, the variables included in the CAMHM are better at explaining current NHPCC usage in model 2 (NHPCC in comparison to past NHP usage) than in model 1 (NHPCC in comparison to nCC-NHP).

Turning to the predisposing factors, the share of females was significantly higher among current NHP users (NHPCC and nCC-NHP users) than among past NHP users. This finding is in line with previous studies [4, 70–72].

The share of elderly people was significantly lower in the NHPCC user group than in the other groups. By analogy, regression model 1 showed that the probability of being an NHPCC user decreased significantly compared to nCC-NHP users with a higher age. The need factor of having more difficulties in daily attention and memory performance significantly increased the likelihood of NHPCC usage compared to nCC-NHP usage and past NHP usage. Taking the predisposing factors of “being female” and “children in the household”, which both had significantly higher shares among NHPCC users than among past NHP users in the bivariate analyses, and the specific need factor DAMP in combination, NHPCC usage could be linked to demanding life circumstances, for example, the balance between work and family, that often affect women [73, 74]. Stress [75] is a common issue in this lifetime, often affecting concentration and cognition, for example, due to sleep issues [76] or migraine [62]. Suffering from migraine symptoms, which can impact concentration and cognition, can support NHPCC usage [54, 62, 77]. Previous studies have discussed findings related to the usage of cognitive enhancers in healthy adults for reducing perceived concentration and cognition issues in stress situations [78, 79].

In the bivariate analyses, the openness-to-change value orientation was significantly weaker for past NHP users than for current NHP users (nCC-NHP and NHPCC). This finding strengthens the assumption also found in other studies that openness is a personal characteristic of individuals who regularly use CAM [80–82]. Self-transcendence was a significant indicator for NHPCC usage as distinguished from past NHP usage. A connection could be built here for application in the field of concentration and cognition support. Moral improvement needs cognitive capacities, which are essential for self-reflection and individual talent development that can themselves be motivators for cognitive enhancement [83, 84]. Another study found self-transcendence to explain cognitive ability in the elderly [85]. Motivated by self-transcendence-orientated values like universalism and benevolence, the cognitive improvement of one person can also benefit the wellbeing of others when it is wanted and used in an adequate manner [84].

For the general sample of NHP-using participants, our study found self-transcendence to be the strongest value orientation, followed by conservation and openness-to-change value orientations. Self-enhancement was the weakest one. This relation in the strength of value orientations among NHP users is consistent with that of the general German population [86].

Taking HSU factors into account, aiming NHP usage on health support and illness prevention were indicators for NHPCC usage as distinguished from nCC-NHP and past NHP usage. Previous studies have found that individuals with difficult to diagnose sicknesses or very subjective health issues are especially inclined to use NHP [72]. For example, individuals with migraine were seeking more professional advice about NHP than, for example, individuals with diabetes [77]. Current NHP users took NHP significantly more often in self-medication than past users did, and self-medication significantly increased the likelihood of NHPCC usage compared to past NHP usage. Overall, self-medication plays an important role in NHP usage [87]. Concerning the enabling factors, health professionals were the most often mentioned NHP information source and an indicator for NHPCC usage. This finding supports the results of previous studies, suggesting the important role of health professionals, including pharmacists, in the healthcare system due to information distribution [30, 31, 88]. By providing professional health advice, including risk and safety information, pharmacists can encourage informed self-medication and informed and safer decision making for NHP. Therefore, evidence-based information sources and adequate educational training for health professionals is necessary to enable them to provide accurate NHP counseling [30, 89, 90]. The perceived knowledge about NHP can be very different among users. A previous study found that women, in particular, with a medium level of self-perceived knowledge about HM were interested in more information [91]. Specific information is necessary if the medical plant used or the targeted application field is not well known by consumers or patients [92]. For example, this could be the case for new NHPCC, which could contain neuroactive hop-extracts as shown by recent studies [92–95]. NHP information seeking via literature/journals was another significant indicator for NHPCC usage compared to nCC-NHP or past NHP usage. One reason could be the specific indication field of CC, which is popular, for example, among students [96–98], who are familiar with scientific literature and journals due to their academic environment. The low rate of Internet utilization to seek information in the study at hand stands in contrast to a study from 2018, which found the Internet to be the most frequent information source for HM in Germany [4] and to another study which found online sources as a medium more often used for health information seeking during the Covid-19 pandemic [99]. However, health information behavior might have changed due to the COVID-19 pandemic and related restrictions [100–102]. Dreisiebner et al. 2021 found that health information sources were used differently depending on sociodemographic characteristics. For example, higher educated people used fewer online communication with friends, and older people used less social media and streaming services but more newspapers [101]. Future research is needed, to get further insights into NHP information seeking via the internet.

Targeted information and communication campaigns by private and public actors are not only necessary for specific NHPCC applications but also for general use of NHP and related implications [103]. Healthcare facilities, such as medical practitioners and hospitals that are authorized to deal with serious health issues, can be unburdened from dealing with non-fatal health issues when individuals use the option to gather information themselves via other channels. This perception has recently been strengthened by worldwide studies examining the importance of pharmacies during the COVID-19 pandemic [100, 104–106].

Finally, we address the strengths and limitations of our study. There are few studies in the literature that apply the CAMHM. One study using the CAMHM did a comparative analysis of CAM use in non-cancer and cancer populations in the United States of America, including different CAM practices and products in their analyses [52]. Another study among African Americans examined CAM use by modifying the AHUM by adding the factor block of disease states. Next to some predisposing, enabling, and need variables, some disease state variables, such as migraine or recurring pain, were found to be related to CAM use [54]. However, most previous studies did not report R² values. For better comparisons and a detailed discussion, we recommend that future studies report R² values.

One asset of our study is that we applied the CAMHM framework with the same set of variables on general NHP usage as well as on NHP usage for a specific indication. Even if some predisposing and enabling factors are commonly included in most studies (e.g., age, gender, occupation), differences can be found, especially for the inclusion of need factors, that can be associated with specific indication fields like cancer or anxiety [46]. We could not find previous studies that tested a model with the same variable set on general and specific NHP usage. Therefore, this study provides valuable insights into the use of NHP and the related predisposing, enabling, need, and health service factors. Future research could test the CAMHM for other specific indications of NHP because indicators differ, for example, between acute and chronic diseases [107].

Due to the online panel sampling, there is a certain risk of bias within this study [108]. First, conducting an online survey excludes individuals without Internet access. However, in 2022, 95% of the German population used the Internet [109]. Second, even with our careful data screening to ensure data quality, recall bias in the answers of participants could not be entirely eliminated. Recall bias are a potential source of bias in the statistical analyses due to incorrect classifications, leading to biased prevalence rates. Even though participation did not require an interest in NHP, the sample could be biased towards individuals who are generally attracted by the topic of NHP and willing to report on their current use. Additionally, answers could be biased toward a perceived socially expected direction. It is possible that participants had health products in mind that were outside the definition of NHP in this study. To avoid such misunderstandings as much as possible, the questionnaire listed, for example, subgroups of NHP within our definition. However, the literature holds different definitions of NHP, which might limit direct comparisons of studies. Further, the results of this study are not likely to be transferable to other nations, especially concerning values, which differ among cultures [86, 110, 111]. But other factors, for example, the healthcare systems, insurance schemes, and health policies, also differ among countries and could influence corresponding results [33, 72]. Comparable studies conducted in other countries can contribute to knowledge about fundamental differences in and influences on health-related decision making by individual consumers in differently developed economies [33].

## Conclusion

The widespread use of NHP in Germany shows their significance in the German healthcare system. The findings of this study give insights on the extent of predisposing, enabling, need, and health service use factors within different NHP usage groups. It also examined which factors indicate NHPCC usage as distinguished from nCC-NHP and past NHP usage.

Health professionals and policymakers should be aware of the different factors that affect NHP and NHPCC consumption and consider them in the development and optimization of healthcare and communication strategies. For example, pharmacists should be trained and be given easy access to up-to-date knowledge about NHPs to enable them to consult with NHP users in an evidence-based, engaging manner. NHP manufacturers should be motivated to optimize their on-product communication with the consumer by providing information in a transparent and easily comprehensible way as well as, for example, to consider specific information on NHPCC in literature and journals. This group can consider the insights of this study to fine-tune their product and communication strategy related, in particular, to specific target groups of NHPCC, taking into account the identified factors facilitating their use. The current study can contribute to characterize the target group of such campaigns and to define the aims and communication channels of such campaigns.

### Electronic supplementary material

Below is the link to the electronic supplementary material.

Supplementary Material 1

## Data Availability

The datasets used and/or analyzed during the current study are available from the corresponding author on reasonable request.

## Abbreviations

NHP: Natural health product
HM: Herbal medicine
CAM: Complementary and alternative medicine
NHPCC: Natural health product for concentration and cognition
OTC: Over the counter
AHUM: Andersen’s healthcare utilization model
CAMHM: CAM Healthcare Model
HSU: Health service use
SSVS: Short Schwartz value survey
APSA: Attention and Performance Self-Assessment
nCC-NHP: NHP not for concentration and cognition indications
SD: Standard deviation
DAMP: Daily attention and memory performance
