# Supplementary material for: Factors influencing the use of natural health products, in particular for concentration and cognition in Germany
Source: BMC Complement Med Ther. 2024 Feb 27;24:103. doi: 10.1186/s12906-024-04407-3 (PMC10898047; doi:10.1186/s12906-024-04407-3)
Supplement: Supplementary file 1 — Supplementary Material 1 [file 12906_2024_4407_MOESM1_ESM.docx]

**Supplementary file 1: Excerpt from the questionnaire**

Questions relevant for the Study *“Usage of Natural Health Products (NHPs) for Respiratory Diseases: User Characteristics and NHP-Consumption Behavior during the Covid-19 Pandemic in Germany”*

**Quotation-Questions**

How old are you

- 18-29
- 30-39
- 40-49
- 50-59
- 60 years +

What is your gender?

- male
- female
- divers

In which federal state is your residence?

- o Bavaria
- o Baden-Wuerttemberg
- o Berlin
- o Brandenburg
- o Bremen
- o Hamburg
- o Hesse
- o Mecklenburg-Western Pomerania
- o Lower Saxony
- o North Rhine-Westphalia
- o Rhineland-Palatinate
- o Saarland
- o Saxony
- o Saxony-Anhalt
- o Schleswig-Holstein
- o Thuringia

How many people live in your residence town?

- less than 5.000
- 5.000 to <20.000
- 20.000 to <100.000
- 100.000 to <500.000
- 500.00 or more

**PART 1: NHP consumption**

1. Which natural health products have you ever used? (multiple answers allowed)

- prescriptive herbal medicine
- over-the-counter herbal medicine
- foods declared as supporting health (e.g. juices)
- natural nutritional supplements
- herbal homeopathic remedies
- other: _______________
- I have never used natural health products. *(exclusive answer/no other answer possible 🡪 skip to part 2)*

2. Have you used natural health products **during the past 12 months** for your own health or illness?

Natural health products include all products that are made from natural ingredients and aim to have a positive impact on health and well-being. These include herbal medicines, natural nutrition supplements and preparations from plants such as health teas.

- yes
- no *(if no skip question 4)*

3. For which of the following health problems have you **ever** used natural health products to treat diseases/symptoms or support your health? (multiple answers allowed)

- Common cold/flu infection
- Covid-19
- Cough
- Insomnia
- Anxiety/restlessness
- Depression
- Concentration/Cognition
- Dementia
- Headaches/migraines
- Pain
- Tinnitus
- Blood pressure
- other: ____________

4. For which of the following health problems have you used natural health products **in the last 12 months** to treat diseases/symptoms or support your health? (multiple answers allowed)

- Common cold/flu infection
- Covid-19
- Cough
- Insomnia
- Anxiety/restlessness
- Depression
- Concentration/Cognition
- Dementia
- Headaches/migraines
- Pain
- Tinnitus
- Blood pressure
- other: ____________

5. Which of the following is/are your goal/s for the consumption of natural health products? (multiple answers allowed)

- Health support/ maintenance
- Disease prevention
- Diseases/symptom treatment

6. Have you also used natural health products in self-medication in the last 12 months without prescription or recommendation by a physician?

- yes
- no

7. Where have you obtained natural health products within the previous 12 months? (multiple answers allowed)

- Pharmacy
- Drugstore (e.g. dm, Rossmann, Müller)
- Selfgrowth or self-collection
- Internet/Onlineshops
- Familiy/Friends
- other: _____________

8. Where do you inform yourself about the effectiveness and possible areas of application of natural health products? (multiple answers allowed)

- General practitioner
- Alternative practitioner
- Pharmacist
- By trial and error
- On product
- Literature/journal
- Family/Friends
- Online/Social Media
- other: __________

===========================

**PART 2:**

**Health Behavior**

In the following section, you will now be asked questions about your general health and health behavior.

Please answer all questions conscientiously and honestly. There are no right or wrong answers. All data in this survey is collected anonymously, which means that no conclusions can be drawn about you personally.

How do you rate your personal health status?

- Very good
- Good
- Medium
- Poor
- very poor

Do you suffer from one or several long-lasting (more than 6 months) health issues or chronic diseases?

(chronic diseases are long-lasting diseases, that require ongoing treatment and medical control, e.g. diabetes, osteoarthritis, migraine, hay fever, asthma, irritable bowel syndrome, mental illnesses, ADD/ADHD)

- yes
- no

**Attention and Performance Self-Assessment (APSA)**

The following questions are about situations that can happen to everyone, or minor mistakes that can happen to anyone from time to time. Some may happen more often than others. We would like to know how often something like this has happened to you in the last 4 weeks. Please select the answer (never - rarely - sometimes - often - always) that applies best to you. Please answer each question and do not leave any question blank.

| Statement | never | rarely | sometimes | often | always |
| --- | --- | --- | --- | --- | --- |
| I started with one thing and suddenly did something completely different without meaning to. |  |  |  |  |  |
| Even a small noise from the surroundings could disturb my reading. |  |  |  |  |  |
| I made a mistake because I wasn't trying hard enough. |  |  |  |  |  |
| I was only able to concentrate for a very short time. |  |  |  |  |  |
| Sometimes I couldn't think of something even though it was on the tip of my tongue. |  |  |  |  |  |
| I had difficulty following a conversation when more than one person was involved. |  |  |  |  |  |
| I daydreamed when I was supposed to be listening to someone. |  |  |  |  |  |
| Noises in the environment distracted me more easily when I was tired. |  |  |  |  |  |
| I don't even need to start work if something is bothering me. |  |  |  |  |  |
| I became impatient with my work. |  |  |  |  |  |
| I acted differently than I had originally planned. |  |  |  |  |  |
| There were occasions when I suddenly didn't know what I wanted to do. |  |  |  |  |  |
| I made little mistakes when I was tired. |  |  |  |  |  |
| I forgot appointments. |  |  |  |  |  |
| I could find something that I had put away a few days before. |  |  |  |  |  |
| I had to go back to my home to get something I had forgotten. |  |  |  |  |  |
| I didn't catch everything during conversations because people spoke so quickly. |  |  |  |  |  |
| I had to read a newspaper article several times to get the context. |  |  |  |  |  |
| After using a word, I asked myself if I had used it correctly. |  |  |  |  |  |
| My mind wandered when I should have been concentrating on something. |  |  |  |  |  |

**Short Schwartz Value Survey (SSVS)**

Please read through the following values and their descriptions. Then tick how important these values are for you personally.

| Value | Against my values | Not important at all | Not important | Rather not important | Rather important | important | Of supreme importance |
| --- | --- | --- | --- | --- | --- | --- | --- |
| **Power**: social status and prestige, control or dominance over people and resources |  |  |  |  |  |  |  |
| **Achievement:** Personal success through demonstrating competence according to social standards |  |  |  |  |  |  |  |
| **Hedonism:** Pleasure and sensuous gratification for oneself |  |  |  |  |  |  |  |
| **Stimulation:** Excitement, novelty, and challenge in life |  |  |  |  |  |  |  |
| **Self-Direction:** Independent thought and action, choosing, creating, and exploring |  |  |  |  |  |  |  |
| **Universalism:** Understanding, appreciation, tolerance, and protection for the welfare of *all* people and of nature |  |  |  |  |  |  |  |
| **Benevolence:** Preservation and enhancement of the welfare of people with whom one is in frequent personal contact |  |  |  |  |  |  |  |
| **Tradition:** Respect, commitment, and acceptance of the customs and ideas that traditional culture or religion provides |  |  |  |  |  |  |  |
| **Conformity:** The restraint of actions, inclinations, and impulses that are likely to upset or harm others and violate social expectations or norms |  |  |  |  |  |  |  |
| **Security:** Safety, harmony, and stability of society, relationships, and self |  |  |  |  |  |  |  |

**Sociodemographic data**

Wat is your highest educational achievement?

- Without graduation
- Secondary modern school or equivalent
- Graduation from polytechnical school in GDR
- School leaving graduation
- Bachelor
- Diploma
- Master
- Ph.D.
- Alternative degree:______________

How is your occupation status?

I am…

- employed
- Not employed (including pupils or students, job seekers or unemployed, early retirees, pensioners)

How many people live permanently in your household, including yourself?

This includes all persons with whom you live and manage together.

- One person
- More than one person

Counting me in, ___ people are living in my household, including ___ children aged under 18 years.

What is your health insurance?

- Public health insurance
- Private health insurance
